# Primary treatment of painful bone metastases using magnetic resonance guided focused ultrasound – initial clinical experience in Taiwan

**DOI:** 10.1186/2050-5736-3-S1-P2

**Published:** 2015-06-30

**Authors:** Hsin-Lun Lee, Jeng-Fong Chiou, Jo-Ting Tsai, Chia-Chun Kuo, Shiu-Chen Jeng, Gong-Yau Lan, Cheng-Yu Chen

**Affiliations:** 1 Taipei Medical University, Taipei, Taiwan; 2 Chang Gung Memorial Hospital, Taipei, Taiwan

## Background/introduction

Magnetic Resonance guided Focused Ultrasound (MRgFUS) has been recently proven and was approved by the FDA as an effective method for pain palliation in patients with painful bone metastases from the US, Europe and Russia. This report describes the first clinical experience in Taiwan.

## Methods

Between May and June 2014, 8 patients suffering from painful bone metastases were identified and recruited by the radiation oncology departments of Taipei Medical University Hospital, Wan Fang Hospital and Shuang Ho Hospital in Taipei, Taiwan. All patients chose to undergo MRgFUS treatment as their first choice of treatment for their painful bone lesions. Treatment was conducted using the ExAblate system (InSightec Ltd.), which integrates a focused ultrasound phased array therapeutic compartment with a magnetic resonance imaging (MRI) scanner (GE 1.5T MRI). Therapeutic ultrasound waves were targeted to the painful lesions while monitoring the accumulated heat using real-time MR images. Efficacy of MRgFUS treatment was evaluated using the numerical rating scale (NRS), before treatment, and at 1 and 3 days; 1 and 2 weeks and at 1 month after treatment. (3 months follow-up results will be collected and reported when available).

## Results and conclusions

The median age of the 8 treated patients (5 male and 3 female) was 58 years old (range, 40 – 76). Each patient underwent treatment of a single lesion.

Majority of treatments were aimed at lesions located in the pelvis (5 sacro-iliac joints and one ilium), one at the femur and one at the sterno-clavicular joint. Three of the targeted lesions were osteoblastic, two osteolytic and three were mixed. None of the patients experienced any procedure related adverse events and the treatment was tolerated well by all. At day 1 post-treatment, 100% of patients reported a significant reduction of pain (median 3.5 points reduction in the NRS, P<0.001) from median baseline NRS of 6 (4-8). By the first month after treatment the median NRS decreased to 3 (range, 1-5, P=0.001). 25% of patients experienced complete response (pain score decreased to 0). The median length of the procedure (patient walk-in to walk-out) was 01:45 hours (01:05 to 02:20 hours), of which the median treatment time was 52 minutes (25-80). The results of these 8 primary treatments are similar to those of Hurwitz et al (2014) which were mainly obtained from radiation failures or patients who could not obtain radiation.

